# Nanostructured Microsphere Production by Osmotic Extraction of Microfluidic Emulsion Templates

**DOI:** 10.1021/acs.langmuir.5c02866

**Published:** 2025-08-06

**Authors:** Kate A. Sanders, Michael F. L. De Volder

**Affiliations:** Department of Engineering, University of Cambridge, 17 Charles Babbage Road, Cambridge CB3 0FS, U.K.

## Abstract

Microscale emulsion droplets are versatile soft templates for assembling nanoparticle powders and forming secondary microparticles. When combined with microfluidics, nanostructured microparticles can be produced with precise size and uniformity. However, assembly requires removal of the droplet solvent, which is particularly slow for water-in-oil emulsions. This has been a longstanding challenge, preventing the deployment of emulsion-structured nanomaterials at scale. Here, an osmotic pressure-driven method is presented that achieves controlled water extraction from emulsion droplets. This is a faster, more cost-effective, and sustainable alternative to prolonged heating; particle solidification is achieved by introducing a second emulsion containing a high solute concentration. The effect of system composition and droplet size on the rate of water extraction, emulsion stability, and nanoparticle assembly is explored, generating an empirical model for the solidification of 100–1000 μm diameter template droplets. By combining this extraction method with microfluidic emulsification, batches of spherical microparticles were formed composed entirely of nanoparticles, in this case, carbon nanotubes as a model system. Particle solidification was up to 5 times faster than evaporation while maintaining control over morphology and size distribution. Additionally, this processing method was demonstrated on other nanoparticle systems, confirming a broad material applicability.

## Graphical abstract

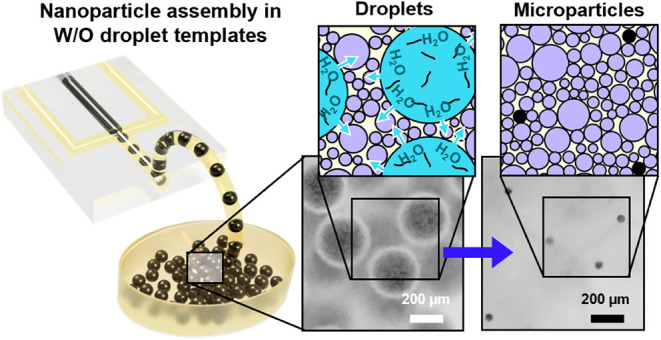

## Introduction

The synthesis of various nanoparticles has been well established over the past decades, and as a result, bulk powders are now available at scale for cost-efficient prices. However, nanomaterial powders may be difficult or hazardous to handle, and the properties of unstructured materials are often disappointing compared to those measured for individual particles. Conventional manufacturing technologies also tend to be poorly suited for nanopowders. Instead, a method of harnessing commercial nanomaterial properties that has attracted significant research attention is to form discrete secondary microscale particles, which can then be assembled into structures or devices with controlled multiscale porosity. For instance, individual carbon nanotubes (CNTs) possess a combination of exceptional properties as a result of their nanoscale size and tubular graphitic structure, including high electrical and thermal conductivities, excellent mechanical strength, and straightforward functionalization.^1^ When compared to unstructured CNT powders, CNT-based microparticles with carefully controlled morphologies have already shown exceptional performance where a high specific surface area, fast mass transport, or low pressure drop are important alongside their intrinsic properties, including for electrochemical sensors,^2,3^ environmental remediation,^4–9^ energy storage,^10–12^ thermal absorption,^13,14^ and catalysis.^15–19^ However, without fast and scalable microparticle production methods, these materials will struggle to reach real-world applications.

A particularly promising route to fabricate microparticles from nanoparticles is through the use of emulsion droplets as templates. This technique can be combined with microfluidic emulsification to generate perfectly monodisperse droplets with adjustable sizes and exceptional control over morphology.^3,7–9,20–22^ The process by which these liquid droplets containing dispersed nanoparticles are then converted to solid particles is a critical and often limiting step. Although polymeric additives may be used, which undergo rapid solidification by thermal,^21,23^ UV light,^21^ or chemical stimuli,^4^ the resulting microparticles usually have a limited nanoparticle content, and often, the end application requires the surface of the nanoparticles to be accessible rather than covered in polymers. Alternatively, the droplet solvent can be removed by drying, compacting the nanoparticle cargo into solid, spherical aggregates by interfacial tension.^3,7,9–11,20,22^ However, many nanoparticles cannot be dispersed in high concentrations in solvents, including CNTs, even after modifications such as oxidation. Substantial volumes of solvent therefore need to be extracted from each droplet before nanoparticle consolidation into a microstructure. Herein lies a significant challenge: the dispersed and continuous phases of an emulsion must be immiscible; therefore, droplet dissolution in the surrounding phase is usually very slow, especially for water-in-oil systems. Microparticle solidification therefore necessitates lengthy drying steps during which droplets tend to coalesce, leading to losses in control over particle diameter and size distribution, and limiting material throughput.

In this work, we describe a method to increase the rate of water loss from water-in-oil emulsions and generate nanostructured microparticles through a novel controlled osmotic extraction process. We demonstrate our process in the fabrication of CNT microparticles, creating regular, spherical aggregates assembled entirely from oxidized carbon nanotubes at rates up to 5 times faster than classic evaporation alone.^20^ The use of an osmotic pressure gradient is an attractive method of achieving fast and uniform solvent transport rates for water-in-oil droplets, as the local extraction conditions can be fine-tuned by adjusting composition, and energy-intensive steps such as heating at high temperatures are not required. However, applying an osmotic gradient at the surface of the microdroplets is not trivial and to date has mostly been achieved through the formation of double emulsions.^24–28,35^ Instead, we propose an alternative approach, which is simpler to implement and has greater flexibility, where we introduce a second emulsion of smaller droplets containing high concentrations of glucose, which surrounds the template droplets. The osmotic extraction of water then occurs through the oil film at the interface between the two droplet phases. We use an inexpensive oil phase investigated previously,^29^ which unlike recent studies does not require perfluorinated solvents or surfactants (which are environmentally persistent and harmful) to stabilize droplets.^30^ Our method shows significant potential for generating nanostructured microparticles from aqueous droplets at higher throughput, maintaining a narrow size distribution, and without substantially increasing process complexity or cost. Additionally, this technique is not material-specific, so it may be extended to further microparticle compositions.

## Experimental Section

### Materials

All chemicals were purchased from Sigma-Aldrich and used as received unless noted here. Nitric acid (HNO_3_, 70%), isopropanol (IPA), and DI water (<5 μS/cm) were obtained from Fisher. Isoparaffin (Isopar G, C10–12) was obtained from ExxonMobil.

### Characterization

A BX53M (Olympus) combined reflected/transmitted light microscope with an LC30 camera was used for optical microscopy. SEM images were taken on a Leo Gemini 1530VP (Zeiss), or a Phenom Pro desktop SEM, with an acceleration voltage of 2–10 kV. Zeta potential measurements were made on a Zetasizer Nano ZSP (Malvern).

### CNT Dispersion Preparation

Multiwalled CNTs (MWNTs, Nanocyl NC7000) were oxidized in a microwave reactor (Anton Paar Multiwave) using concentrated HNO_3_ (20 mL for 100 mg CNTs) at 180 °C for 30 min. The reaction mixture was filtered under vacuum, and the filter cake was washed with DI water until the filtrate was neutral. The resulting ox-CNTs were dried in an oven (80 °C, 24 h) and redispersed in DI water by ultrasonication in a bath sonicator (6 h, < 60 °C).

### Oil Phase Preparation

The oil phase used in this work was composed of a mixture of isoparaffin and oleic acid in a 5:2 volumetric ratio. A surfactant mixture of 4 wt % sorbitan monooleate (Span 80) and 1 wt % ethoxylated sorbitan trioleate (Tween 85) with a total HLB of 5.6 was used for interfacial stabilization. This oil phase composition was chosen as our previous work demonstrated its excellent stability for aqueous droplets in close contact,^29^ and the addition of oleic acid may improve water solubility compared to hydrocarbon oils.

### Draw Emulsion Preparation

Draw solutions were made up in DI water as per the required concentration. If solutes proved difficult to dissolve, the solution was gently heated in a 60 °C water bath and cooled to room temperature before use. Coarse W/O draw emulsions were generated by mixing the required volume of each phase with a vortex mixer at 2500 rpm for 30 s (see Figure S1). Fine W/O draw emulsions were generated by ultrasonication (Misonix S-4000, 20 kHz, using a Microtip probe (1.6 mm diameter, 320 μm amplitude)). Sonication for 30 s at an amplitude of 50% resulted in a uniformly mixed emulsion where individual droplets could not be distinguished by optical microscopy (<1 μm droplet diameter, Figure S6).

### Water Extraction Rate Tests for CNT Template Droplets

After preparation of the selected draw emulsion, 1.5 mL was transferred to a 35 mm diameter Petri dish (interface height of 1.6 mm). Droplets of aqueous ox-CNT dispersion were then generated in the draw emulsion using a micropipette (for droplets *r*_0_ > 200 μm, Figure 4) or by mixing in a few drops of vortexed emulsion (for droplets *r*_0_ < 200 μm, Figure S7). The first image (*t* = 0) was acquired immediately after template droplets contacted the draw emulsion. After the allocated time for extraction, droplets or particles were pipetted onto a glass slide and rinsed with IPA to assess solidification. All experiments were conducted at room temperature (18–20 °C) unless noted. Template droplet and microparticle areas and so radii/diameters were measured using Fiji (ImageJ). Independent of the initial drop size, the relationship between average relative radius and time was approximately linear until the final stages of particle consolidation. The rate of the water transport process was therefore estimated by linear fits for *r*/*r*_0_ ≥ 0.3.

### Microfluidic Monodisperse CNT Microparticle Generation

Flow-focusing microfluidic droplet generators were fabricated from 3D-printed molds by soft lithography from PDMS, as described in our previous work.^7,20^ Aqueous ox-CNT dispersions and draw emulsions were used as the dispersed and continuous phases, with flow rates set at 100 and 400 μL/min, respectively, using syringe pumps (NE-1000, KF Technology). Droplet size varied according to the viscosity of the continuous phase. Emulsified droplets were collected from the device outlet in a dish containing 1.6 mm (height) of the oil phase. Additional experiments were performed by emulsifying template droplets in oil and collecting them in draw emulsions, but mixing was less effective (see Figure S10).

### Polydisperse CNT Microparticle Batches

Aqueous nanoparticle dispersion was added to the oil phase, and the mixture was emulsified with a vortex mixer at 300 rpm for 5 s. Immediately after emulsification, this was mixed with ultrasonicated draw emulsion with a 1:5 (v:v) glucose:oil composition to yield a 1:10 overall draw emulsion composition. Polydisperse microparticles could also be generated by emulsifying template droplets directly in a 1:10 draw emulsion as the continuous phase with comparable results (see Figures S14 and S15), but this couples the emulsification conditions to the draw emulsion properties, which is less desirable.

### Separation and Washing of Solid CNT Microparticles

After particle solidification, the emulsion was diluted to double the original volume with isoparaffin, shaken vigorously, and immediately filtered under reduced pressure. Microparticles were washed on the filter with ethyl acetate to remove residual surfactant, followed by IPA, which was evaporated to give dry particles.

## Results and Discussion

A schematic of the proposed mechanism of microparticle assembly from nanoparticles is shown in Figure 1. As stated in the introduction, CNTs were used as an exemplar nanomaterial building block, which benefit hugely from controlled microscale assembly but are challenging to process by existing methods. Commercial multiwalled CNT powders were first oxidized (denoted as ox-CNTs) in nitric acid, as this facilitates their dispersion in water and provides reactive sites for application-specific functionalization of the resulting microparticles if desired. Aqueous CNT dispersions were then emulsified (e.g., microfluidics) in a mixed hydrocarbon oil phase containing 5 wt % nonionic surfactants. These template droplets were combined with a second emulsion containing a high concentration of solute, which we term the “draw” phase (purple in Figure 1). Flocculation of the smaller draw phase droplets results in a total coverage of template droplet interfaces. The osmotic pressure difference between the two aqueous phases drives local water transport through intermediate oil layers and can be controlled precisely by adjusting the composition. Due to their nanoscale size and hydrophilic functional groups, CNTs remain in the template droplet during extraction and aggregate in a controlled manner as a result of capillary forces and interparticle interactions, forming a spherical microstructure.

**Figure 1:**
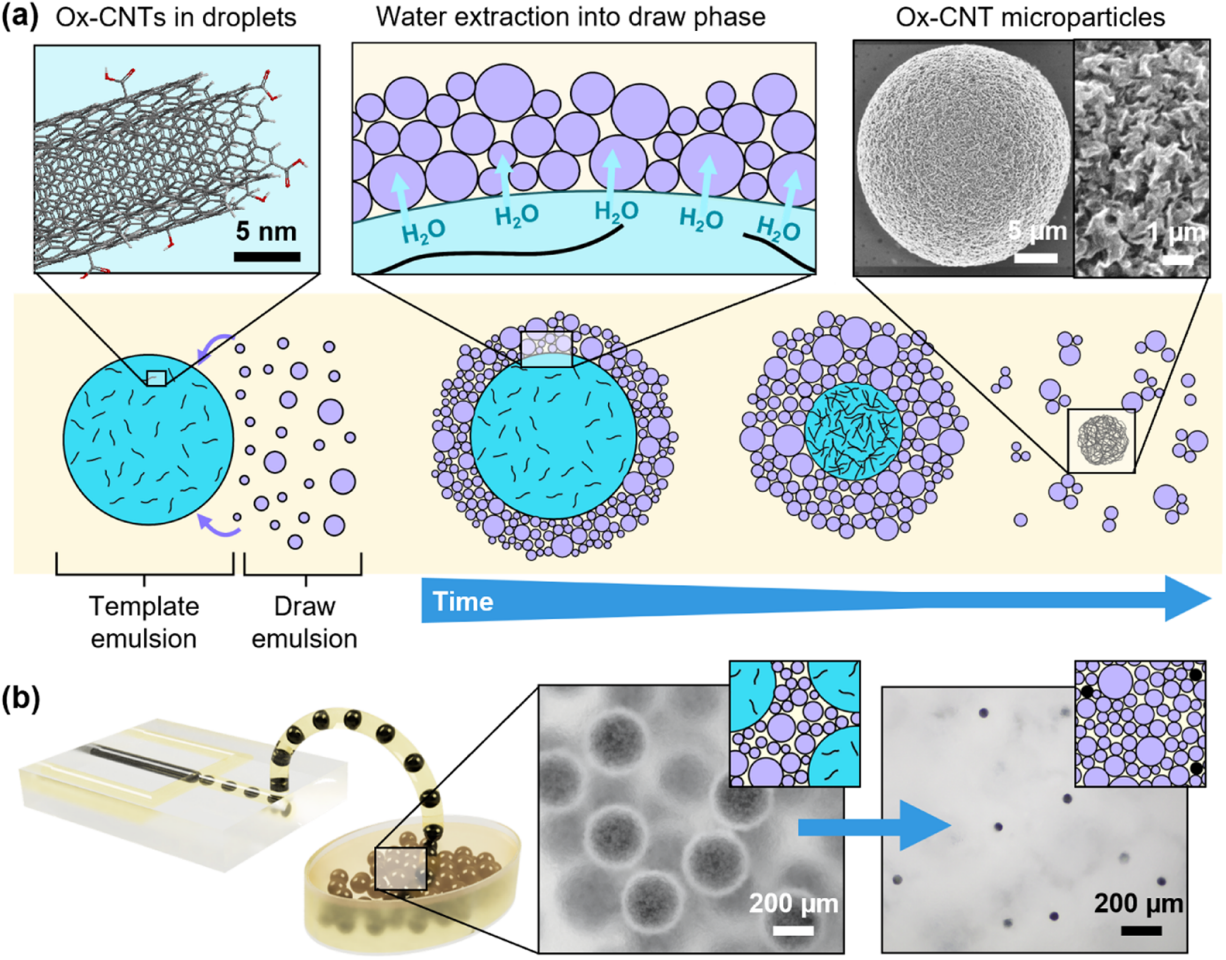
Overview of osmotic pressure-driven microparticle formation from nanoparticles in W/O emulsions. (a) Schematic showing the osmotic pressure-driven process of rapid, selective water extraction from a template droplet (blue) containing dispersed oxidized carbon nanotubes (ox-CNTs) into aqueous draw emulsion droplets containing a high concentration of solute (purple). As water is removed, solid, spherical microparticles form, composed entirely of ox-CNTs. (b) Illustration of collective water extraction from a batch of microfluidic-generated droplets of identical size, showing monodisperse CNT droplets and particles surrounded by draw emulsion.

In this work, we initially establish the key parameters influencing the rate of osmotic extraction, such as draw solute type, concentration, and droplet size, and then demonstrate size-controlled CNT microparticle production using batches of microfluidic-generated and bulk-emulsified droplets as templates. Our method is particularly suitable for collective processing of droplets as the physical barrier provided by smaller draw emulsion droplets reduces template droplet coalescence, so the size distribution from microfluidic emulsification is preserved without the need for specialist surfactants (Figure 1b). Additionally, the rate of water removal depends predominantly on local water transport to the draw phase, so occurs at a uniform rate even for larger volumes of emulsion and, unlike evaporation, does not depend on an air–oil interface.

We can consider water transport between the two aqueous phases in the process proposed in Figure 1 as a forward osmosis (FO) process, with water flux described in its simplest form as *J*_w_ = *P*_w_ΔΠ, where *P*_w_ is the permeability of the intermediate oil layers to water. The “feed” or template droplets containing nanoparticles and the smaller draw droplets containing a solute must therefore maintain a substantial osmotic pressure difference, ΔΠ = Π_draw_ – Π_feed_. Although a high concentration of solute in the draw phase will increase ΔΠ, one of the key challenges for osmotically driven processes is reverse solute flux (RSF), which occurs from draw to feed droplets and also increases with solute concentration.^31,32^ High concentrations of dissolved solutes in emulsions may also affect surfactant solvation and interfacial tension, which can increase the rate of breakdown processes.^33,34^ Additionally, despite our aim of achieving rapid particle formation, fast mass transport and droplet swelling in emulsions have been associated with decreased stability and interfacial rupture.^34–36^ Progressive RSF or coalescence of droplets in the emulsion are both likely to inhibit solid microparticle formation, as the contamination of template droplets by draw solute may affect CNT assembly and limit droplet volumetric shrinkage.

Previous studies of mass transport between aqueous phases in emulsions have shown that the movement of species across oil and surfactant regions can take place through dissolution and diffusion or surfactant-assisted processes, and multiple mechanisms may take place simultaneously. For aqueous phases in close proximity or with direct interfacial contact, water transport in the presence of an osmotic pressure gradient is thought to be dominated by direct diffusion or molecular surfactant hydration.^35–42^ Across larger separation distances (∼10s of μm or greater,^38^ water may also be exchanged via reverse micelles or spontaneously formed carrier species.^36–38,40,41,43–45^ In addition to the influence of osmotic pressure, water transport rates tend to increase with a higher surfactant concentration,^36–40^ a more polar oil phase,^24^ more hydrophilic surfactant(s),^36,46^ and decreased oil layer thickness.^24,34,36,46^ Solute transport in emulsions can take place independently or alongside water transport mechanisms depending on interactions with the oil and surfactant(s).^41,47–54^ Despite poor solubility in most oils, inorganic ions can and do move between aqueous phases, with transport rates increasing in the presence of more hydrophilic surfactants,^39^ and for less hydrated ions, although complexation may disrupt this trend.^47,50–52^ Similarly, molecular solute transport across an oil phase tends to increase with decreased molecular weight^48,49,53^ and increasing hydrophobicity or favorable interactions with components in the oil phase.^35,53,54^ Even with substantial previous literature, it remains difficult to decouple the influences on transport processes due to the variation in studied emulsion structures and compositions together with the complexity and chemical dependence of transport. As a result, the suitability of a particular draw solute for water extraction and particle solidification is challenging to predict and has never been thoroughly investigated for W/O emulsions containing nanoparticles. We therefore first performed a comparative study of prospective solutes in the presence of CNT-containing template droplets.

### Draw Solute Extraction Tests

Comparative water extraction tests were carried out with draw solute candidates selected considering high aqueous solubility, low cost, and previous use^32,55,56^ in forward osmosis processes: sodium chloride, glucose, sucrose, fructose, glycerol, and urea. Initial draw emulsions were made by vortex mixing of aqueous draw solutions under the same conditions (see the section titled Experimental Section) using a draw solution:oil phase volume ratio of 1:10 (v:v). The resulting coarse draw emulsions were polydisperse, with diameters between 10 and 100 μm (Figure S1). Large template droplets (initial radius, *r*_0_ = 500 μm) containing ox-CNTs were then generated in these emulsions. These template droplets represent an extreme case to test water extraction due to their large size: a volumetric shrinkage of around 99% before microparticle consolidation is expected for 0.1 wt % ox-CNT/H_2_O dispersions.^7,20^ Template droplet size was monitored over 2 h by dark field (DF) optical microscopy, with any coalescence noted for a qualitative comparison of emulsion stability. A final measurement was taken after 24 h before extracting samples from the emulsion to determine whether microparticle solidification had occurred. As a control, CNT-containing template droplets of the same size were also emulsified in a DI water emulsion containing no draw solute; these droplets swelled very slightly over the same time period (Figure S2).

Representative optical micrographs and shrinkage rates for each draw solute are shown in Figure 2, with further images in Figure S3 and comparative solution properties in Table S1. Immediately after emulsification of the large template droplet, the smaller draw droplets flocculated around its interface and remained in interfacial contact during the subsequent transport of water until solidification. No evidence of spontaneous emulsification was observed at the interface of any draw emulsion composition, likely due to the high concentrations of solute, which reduces the driving force for water transport through this mechanism.^29,38,46,53,54^ The exchange of water and/or solutes through diffusion and molecular surfactant-assisted processes is therefore the most likely mechanism in operation between droplets, although this was not investigated directly. Instead, considering our aim of microparticle formation, the draw solute performance was assessed on the basis of three main factors: droplet shrinkage rate, emulsion coalescence, and whether solid microparticles could be formed.

**Figure 2:**
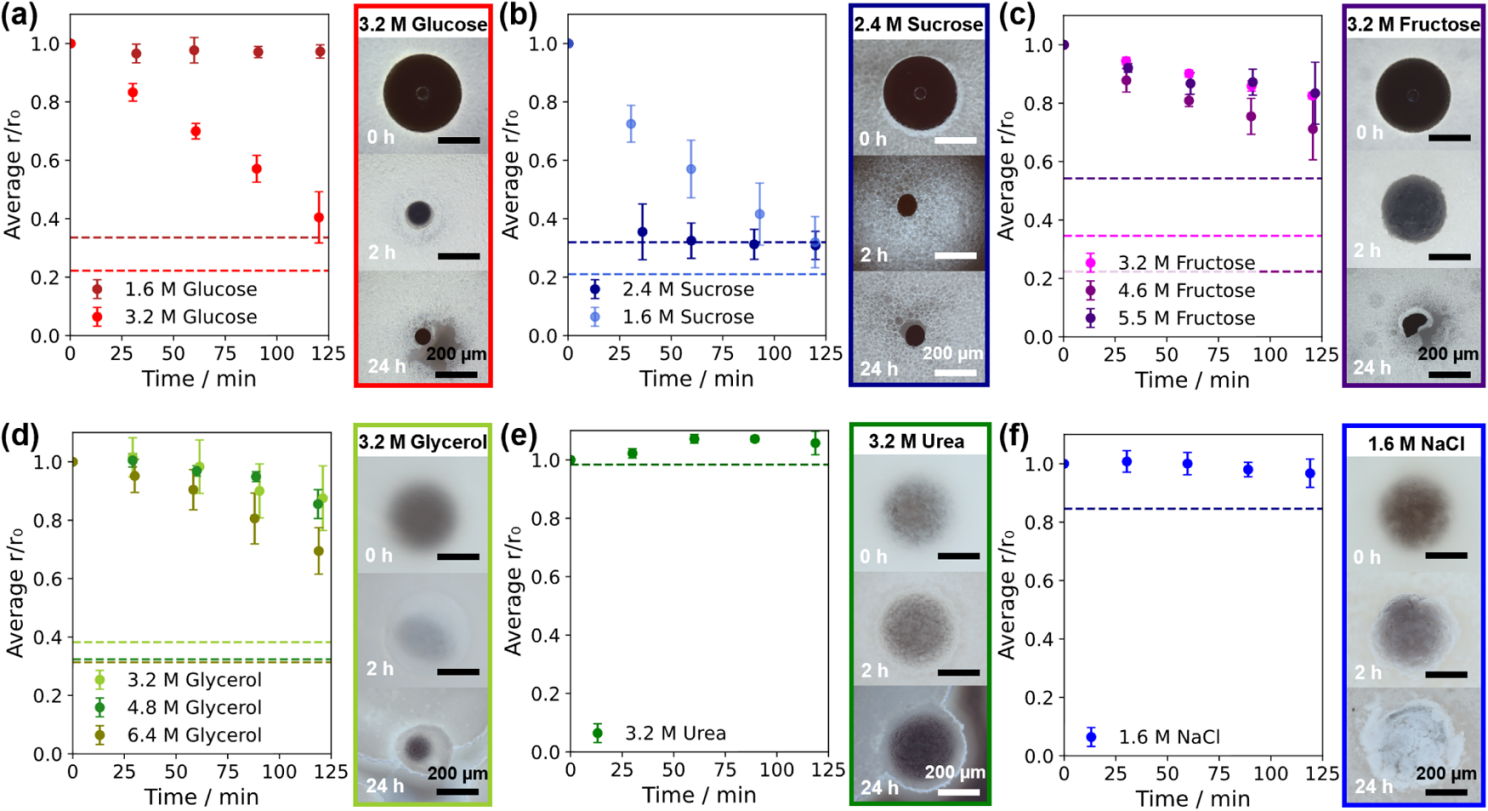
Effect of draw emulsion composition on the water extraction rate from template droplets containing 0.1 wt % ox-CNTs. Draw solutions were (a) 1.6–3.2 M glucose; (b) 1.6–2.4 M sucrose; (c) 3.2–5.5 M fructose; (d) 3.2–6.4 M glycerol; (e) 3.2 M urea; (f) 1.6 M NaCl. A 1:10 volumetric ratio of glucose: oil phases was used. The change in the average relative template droplet radius (*r*/*r*_0_, *r*_0_ = 500 μm, *n* = 3) is shown on the left, with dashed lines indicating average *r*/*r*_0_ after 24 h. Representative optical micrographs are shown on the right. Draw droplet flocculation is visible in the emulsions in (b–e), depleting regions near the initial template droplet location.

The best-performing draw solutes in coarse emulsions were 3.2 M glucose (Figure 2a) and 1.6 M sucrose (Figure 2b), which were both able to form solid microparticles (see Figure S4) and had a rapid average diameter shrinkage of around 5 μm s^–1^ over the first 2 h (d­(*r*/*r*_0_)/d*t* of 0.048 and 0.055 s^–1^ respectively). This was based on fits of initial droplet shrinkage, which were linear for *r*/*r*_0_ > 0.3, as seen in previous studies of W/O or W/O/W droplets shrinking through water extraction into a higher osmotic pressure phase in volumetric excess.^38,40,41^ A higher concentration of 2.4 M sucrose resulted in an even faster rate of water extraction, achieving *r*/*r*_0_ = 0.36 after only 30 min, but draw and template droplet coalescence was observed, and water transport from the template droplet stopped before particles could solidify. Fructose draw emulsions also displayed draw and template droplet coalescence at a 5.5 M concentration, but lower concentrations were slower to extract water than glucose or sucrose (Figure 2c). Glycerol draw emulsions (Figure 2d) showed a similarly slow water extraction rate and could only generate flattened and sticky CNT microparticles after 24 h (see Figure S4), most likely due to reverse solute flux and the liquid state of any residual glycerol. Draw emulsions containing 3.2 M urea (Figure 2e) also performed poorly, with no change in droplet size after 24 h despite no obvious destabilization, again suggesting fast reverse solute transport. The only ionic draw solute tested was 1.6 M sodium chloride, which has the same osmolarity as the 3.2 M organic draw solutes. However, template droplets in this draw emulsion shrank only slightly (Figure 2f), and large, collapsed CNT aggregates were recovered after 24 h rather than compact microparticles. Ox-CNT dispersions aggregated immediately upon contact with the NaCl draw solution (shown in Figure S4), so this result was likely caused by RSF. Like many nanoparticle dispersions, ox-CNTs in water rely on charge stabilization (zeta potential measured at −65 ± 12 mV, Figure S5), so their stability is strongly affected by charge screening effects, which then exclude most of the common salts used in FO processes.^38,40^

Although 1.6 M fructose and 3.2 M glucose had similar water extraction rates in the above tests, a lower draw solute concentration may limit the initial nanoparticle dispersion concentration in the template droplet, so 3.2 M glucose was selected as the most promising draw solute candidate for further study. The effect of changing the CNT concentration in the template droplet composition was then explored (Figure 3): up to 0.5 wt % ox-CNTs could be dispersed in water without further additives. The initial rate of droplet shrinkage containing 0.05 and 0.5 wt % ox-CNTs proved to be extremely similar to 0.1 wt % ox-CNTs (Figure 3a), forming solid microparticles with a concentration-dependent size (*r*/*r*_0_ = 0.15 and 0.3 for 0.05 and 0.5 wt % ox-CNTs, respectively). As a control, pure water “template” droplets were emulsified in a 3.2 M glucose draw emulsion (with 0.025 wt % methylene blue for draw phase contrast). Figure 3b shows similar initial shrinkage rates to CNT-containing droplets up to 60 min, but after this, DI water droplets shrank faster and eventually disappeared entirely. This result suggests the water transport rate is initially dominated by the draw emulsion properties at these dilute nanoparticle concentrations. It is likely that ox-CNTs decrease water transport rates compared to pure water droplets by progressively lowering ΔΠ as they concentrate, as reported for other solutes,^24,30,38,40^ although local nanoparticle concentration polarization near the shrinking interface may also occur. Sequential radial transport of water from pure water droplets through the draw emulsion could also be visualized optically, as the oil/aqueous glucose refractive indices are initially similar but become mismatched upon dilution; the images in Figure 3b imply that the entire draw emulsion participates in solvent extraction of an isolated template droplet.

**Figure 3:**
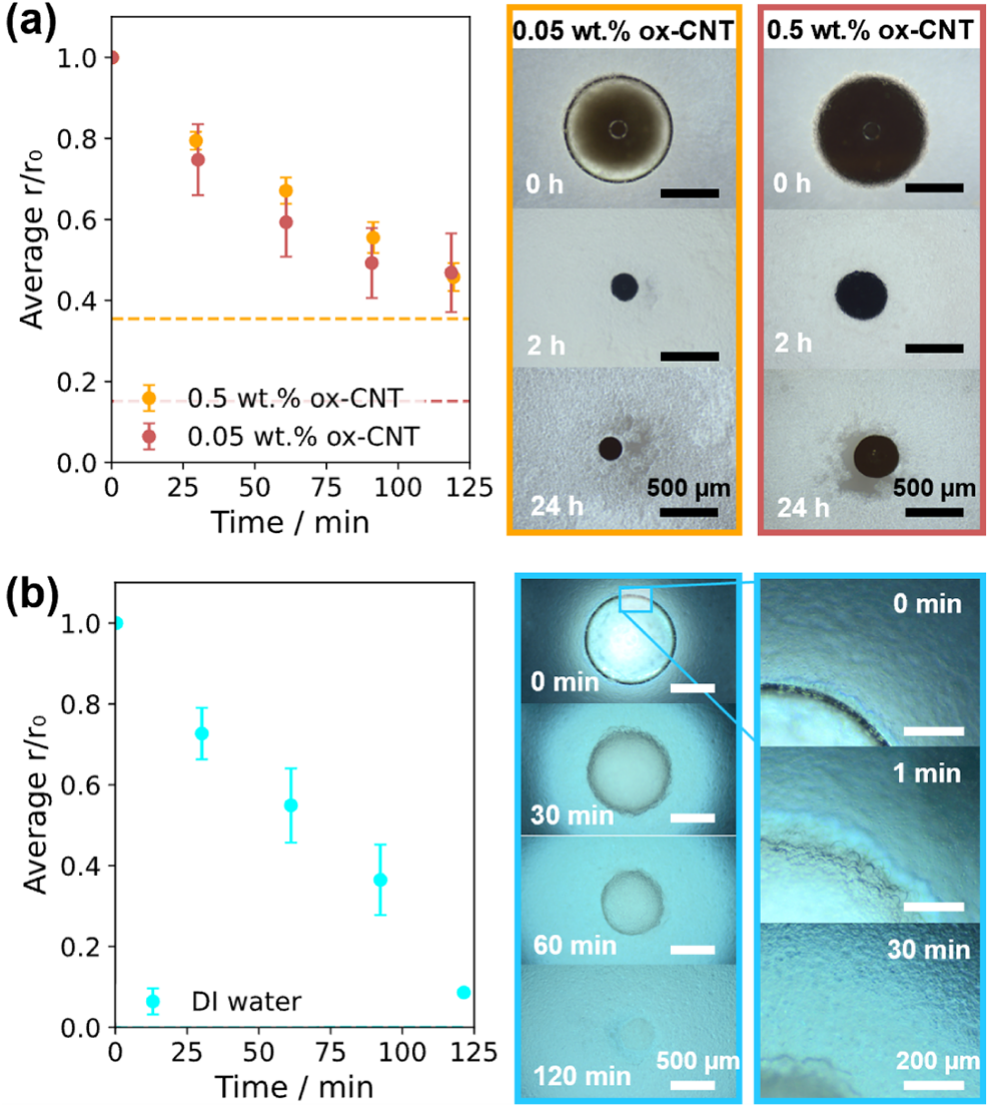
Effect of aqueous template droplet composition on water extraction into 3.2 M glucose draw emulsions. Template droplets contained (a) 0.05 and 0.5 wt % ox-CNTs; (b) DI water. 0.025 wt % methylene blue was added to the glucose phase in (b) for contrast. The change in the average relative template droplet radius (*r*/*r*_0_, *r*_0_ = 500 μm, *n* = 3) is shown on the left, with dashed lines indicating average *r*/*r*_0_ after 24 h. Representative optical micrographs are shown on the right. The last data point in (b) has no standard deviation, as only one droplet remained.

### Factors Affecting Water Extraction Rate

Despite rapid water extraction rates, many of the microparticles generated with coarse draw emulsions had rough surfaces and irregular morphologies (examples in Figure S4). This is likely influenced by polydisperse draw emulsion droplets causing nonuniform extraction and imprinting on the surface in the last stages of nanoparticle consolidation. For instance, Figure 4a shows an SEM image of a representative CNT microparticle, which has surface features of a comparable size to those of the draw droplets. A fine draw emulsion of the same composition was therefore produced by tip sonication, decreasing droplet size to ≤1 μm (Figure S6). This emulsion consistently generated microparticles with smaller surface features (Figure 4b). However, decreasing the droplet size also increases water transport resistance. To quantify the water extraction rate for the fine draw emulsion, a series of CNT-containing template droplets with radii between 50 and 500 μm were studied (further details in the section titled Experimental Section and Figure S7). Rings of different scattering intensities were observed, implying greater dilution and lowering of the local osmotic pressure gradient compared to coarse draw emulsions (e.g., Figure 4c vs Figure 3b). Figure 4d,e shows average template droplet shrinkage rates for 0.1 and 0.5 wt % ox-CNTs, respectively. The droplet shrinkage rate, d­(*r*/*r*_0_)/d*t*, depends critically on initial template droplet size (Figure 4f): smaller droplets have both a lower volume and larger relative surface area, which both favor faster extraction. The smallest droplets, *r*_0_ = 50 μm, took less than 2 h to reach their terminal size: for comparison, droplets with a similar average size (Figure S8) generated in the same oil phase remained liquid even after 6.5 h, illustrating the extremely slow rates of solvent loss at room temperature and the need for alternative methods. From experimental measurements of volumetric shrinkage for the droplet-to-microparticle transition, a solidification time, *t*_s_, was also estimated, which can be fitted to a similar power law for both CNT concentrations (Figure 4g) to predict the microparticle consolidation time within a broad droplet size range.

**Figure 4:**
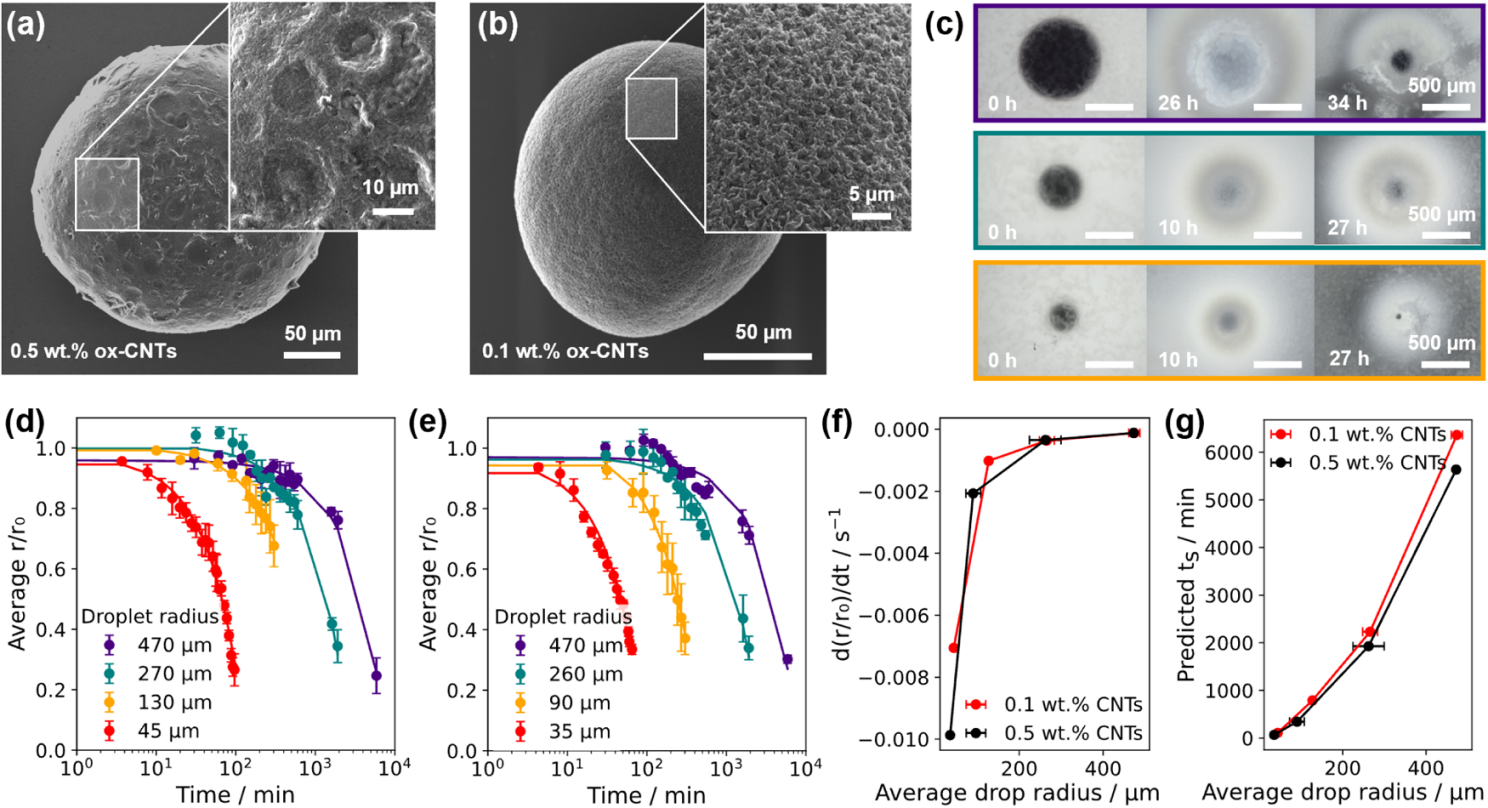
Effects of template and draw droplet sizes on osmotic extraction. SEM images of representative ox-CNT microparticles produced by extraction into draw emulsions generated by (a) vortex mixing and (b) high-energy ultrasonication. (c) Representative optical micrographs of 0.1 wt % ox-CNT/H_2_O template droplets with different sizes undergoing osmotic extraction in the draw emulsion used in (b). Average relative template droplet radius (*r*/*r*_0_, *n* ≥ 3) in ultrasonicated draw emulsions for various *r*_0_, where the initial ox-CNT concentration was (d) 0.1 wt % and (e) 0.5 wt %. Solid lines are linear fits for *r*/*r*_0_ *≥* 0.3. (f) The relationship between the relative water transport rate, d­(*r*/*r*_0_)/d*t*, and the initial droplet radius. (g) Predicted solidification time, *t*_s_, vs initial droplet radius. Lines are a power-law fit *y* = *ax* ^*n*^, where *n* = 1.70, *a*_0.1 wt %_ = 0.20, and *a*_0.5 wt %_ = 0.16.

From the above experiments, it is clear that the rate at which draw emulsions can extract water is limited by transport through the resistive oil phase, which is consistent with the previously reported collective behavior of droplets in close contact.^42^ As temperature increases, the rate of water transport by hydrated surfactants has previously been shown to increase,^39^ and the solubility of water in the oil phase will also be higher. Individual template droplets in the ultrasonicated glucose draw emulsion were therefore heated to 60 or 80 °C, which significantly increased the rate of water loss (Figure 5). Compared to the analogous template droplets at room temperature, d­(*r*/*r*_0_)/d*t* was approximately 10 times greater at 60 °C and 20 times greater at 80 °C for both 0.1 wt % CNTs (Figure 5a) and 0.5 wt % CNTs (Figure 5b). However, at 80 °C, the draw emulsion showed substantial destabilization alongside template droplet shrinkage, forming large droplets with a complex internal structure (visible after 5.5 h in Figure 5a,b and magnified in Figure S9), which may be due to local phase inversion. Solid CNT microparticles could not be extracted, although, considering the results in Figure 4, it is possible that further optimization using smaller template droplets could generate microparticles at 80 °C before emulsion breakdown.

**Figure 5:**
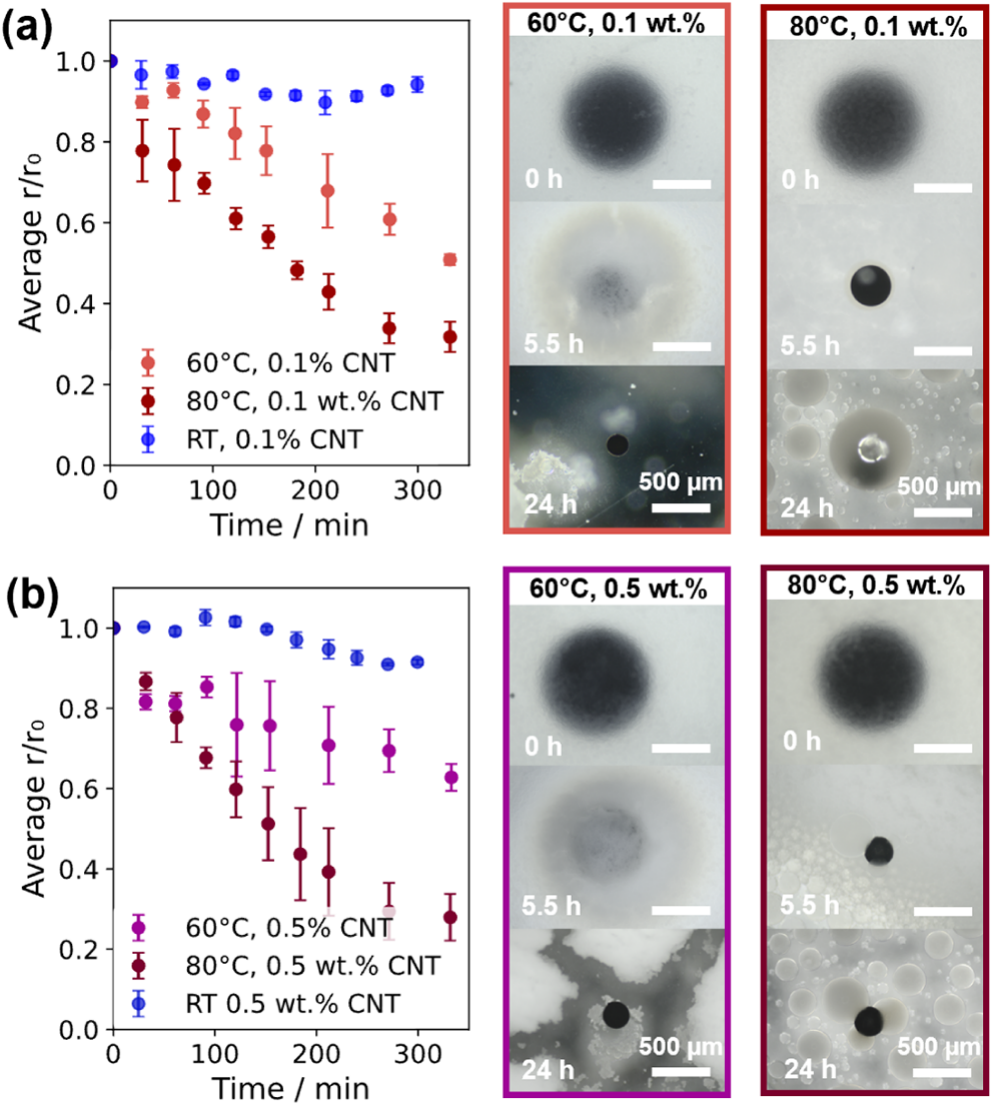
Effects of heating on osmotic extraction into ultrasonicated draw emulsions. Template droplets contained (a) 0.1 and (b) 0.5 wt % ox-CNTs, which were heated at 60 or 80 °C. The change in the average relative template droplet radius (*r*/*r*_0_, *r*_0_ = 500 μm, *n* = 3) is shown on the left. Representative optical micrographs are shown on the right.

### Batch CNT Microparticle Formation

In the final part of this study, we focused on the collective solidification of batches of CNT microparticles through osmotic extraction, informed by the single droplet studies. When scaled up, emulsion processing often fails due to droplet coalescence, which is why current studies are limited to small batches of particles, or rely on expensive perfluorinated oils and surfactants, which provide excellent stabilization.^30,57^ However, perfluorinated substances are environmentally persistent and harmful and are in the process of being restricted or banned in the EU. Here, we demonstrate that, in addition to local osmotic extraction, fine draw emulsions with a small drop size prevent coalescence during prolonged drying and allow for multilayer collection and solidification.

To control template droplet size and size distribution, microfluidic droplet generators were used to emulsify 0.1 wt % CNT dispersions (see the section titled Experimental Section) with a constant dispersed phase volume fraction, φ, of 0.2. Several approaches to combining draw emulsions and microfluidic-generated template droplets were explored (see Figure S10), but the most effective proved to be direct emulsification of template droplets in the glucose draw emulsion. The osmotic extraction of droplets in close proximity also requires consideration of the volume of the draw solute, which sets the effective osmotic capacity of the system. We therefore compared two draw solute compositions, 1:10 and 1:4 (v:v) aqueous glucose: oil phase ratios at room temperature and heating at 60 °C.

At room temperature, the 1:4 draw emulsion composition proved to be the most promising for microparticle solidification (Figures 6a and S11). Our predicted *t*_s_ for an isolated droplet with a similar initial size was around 18 h (*r*/*r*_0_ = 0.18): by comparison, this batch of template droplets reached an average *r*/*r*_0_ of around 0.3 after 24 h (Figure 6b), which is only slightly slower. For comparison, our previous work showed batch droplet-to-particle solidification times of up to 10 days at room temperature for a comparable template droplet size.^20^ The size distributions of template droplets remained similar during extraction (Figure 6c), indicating low rates of coalescence and a locally similar extraction environment for each droplet. This is a significant advantage for microparticle production, as dissimilar droplet solidification rates can cause uncertainty in processing time and issues with wet droplets sticking to dry particles.

**Figure 6:**
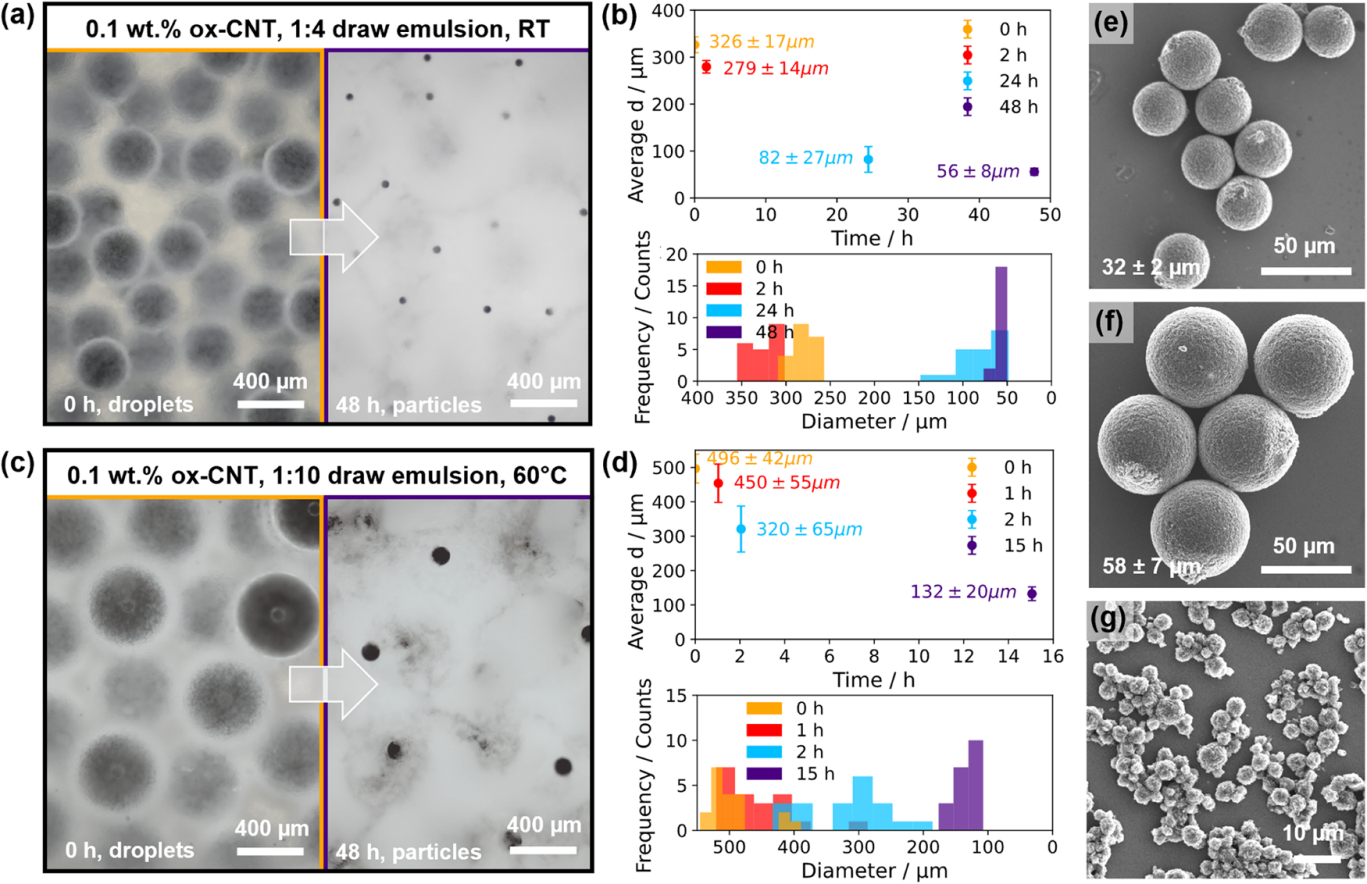
Batch solidification of microfluidic-generated ox-CNT microparticles with ultrasonicated draw emulsions. (a) Optical micrographs of template droplets emulsified in a 1:4 glucose: oil volumetric ratio draw emulsion at room temperature (RT). (b) Average template droplet diameter (top, *n* = 20) and size distribution (bottom) of the emulsion in (a). (c) Optical micrographs of template droplets emulsified in a 1:10 glucose: oil volumetric ratio draw emulsion at 60 °C. (d) Average template droplet diameter (top, *n* = 20) and size distribution (bottom) of the emulsion in (c). (e, f) SEM images of solid ox-CNT microparticles from (a) and (b), respectively. (g) Polydisperse ox-CNT microparticles generated from bulk emulsification after 2 h of osmotic extraction at RT, using comparable conditions to (a).

The solidification of batches of CNT template droplets was also tested at 60 °C, considering our results in Figure 5. Unfortunately, template droplets in the 1:4 ratio draw emulsion began to coalesce before solidification could occur (Figure S12). Both template and draw emulsion coalescence appeared to have occurred, likely due to the combination of a higher dispersed phase volume fraction and elevated temperature. However, the 1:10 draw emulsion composition remained stable over the solidification time scale, and microparticles were generated from ∼500 μm droplets. Despite the large droplet size, the average *r*/*r*_0_ was around 0.26 after only 15 h (Figure 6d). This is significantly faster than the same draw emulsion composition at room temperature (see Figure S11, *r*/*r*_0_ = 0.6 after 24 h) and also compares favorably with our previous work, where similar template droplets required 72 h at 40 °C to fully solidify.^7^ SEM images of microparticles solidified at room temperature (Figure 6e, 32 ± 2 μm (*n* = 46)) or at 60 °C (Figure 6f, 58 ± 7 μm (*n* = 30)) showed regular spherical structures comparable to those generated by evaporation alone (Figure S13). Microparticle size was predominantly controlled by parent droplet size, with a slightly broader size distribution for those solidified at 60 °C (coefficient of variation: 12% compared to 6% at RT).

This work predominantly studies template droplets that are large and contain only small quantities of nanoparticles and therefore represent an extreme example for microparticle formation, which cannot be easily addressed through existing methods. However, importantly, our method of controlled water extraction is not limited to monodisperse emulsions; although microfluidic emulsification can fabricate microparticles of identical size, the same technique is compatible with bulk emulsification. We demonstrated this by fabricating batches of polydisperse CNT microparticles. Using the same volume fraction of nanoparticle dispersion as before, bulk-emulsified template droplets with diameters between 10 and 100 μm solidified in under 2 h to form spherical, regular microparticles with diameters from 2 to 10 μm (examples in Figures 6g and S14 and S15). Similar results were also seen when CNTs were exchanged for dispersions of other commercially available nanoparticles: silica (Figure S16) and metal oxide (Li_4_Ti_5_O_12_, Figure S17) nanoparticles. This clearly demonstrates the versatility of our draw emulsion-based approach, as it does not critically depend on emulsification conditions and nanoparticle composition or require complex tools in order to achieve rapid microparticle formation from nanoparticle assembly in W/O emulsions.

## Conclusions

In this work, we demonstrate that local osmotic pressure-driven extraction in emulsions is an effective method of forming nanostructured microparticles from aqueous droplet templates. Using CNTs as an exemplar nanomaterial, we achieve rapid, selective, and tunable water transport rates. Our processing method operates under mild conditions (room temperature to 60 °C, neutral pH, ambient pressure, no harsh organic solvents) and does not rely on fluorinated oils or surfactants, utilizing only inexpensive components. We established the most effective conditions for microparticle solidification by testing various draw solutes, selecting glucose as the most suitable, and investigating the effect of draw and template droplet size on the water extraction rate. The parameter space for CNT-containing droplets with diameters from 100 to 1000 μm was characterized and empirically fitted to a power law to estimate solidification time.

The same method was then applied to batches of template droplets generated by microfluidics. For large initial droplet diameters and dilute nanoparticle concentrations, microparticle solidification was achieved in 48 h at room temperature and 15 h at 60 °C, generating 30 and 60 μm CNT microspheres. This is around 5 times faster than evaporation while maintaining regular spherical morphologies and a narrow size distribution. Polydisperse bulk emulsions with a size range of less than 100 μm were solidified by the same method in under 2 h to generate CNT particles less than 10 μm in diameter. Importantly, this method of controlled water transport is not specific to CNTs, so it can be used to structure alternative nanoparticle building blocks into microparticles or more generally to manipulate the size of water-in-oil emulsion droplets.

## Supplementary Material



## Data Availability

All data needed to evaluate the conclusions in this work are present in the paper and/or the Supporting Information. Research data underlying this publication are available in Apollo—the University of Cambridge Repository—at 10.17863/CAM.118091.

## Notes

Optical characterization of draw emulsions (Figures S1, S6, and S9); zeta potential of ox-CNTs (Figure S5); control experiments on droplet shrinkage (Figures S2, S8); further characterization of nanoparticle-containing droplets and particles by optical microscopy and SEM (Figures S3–S4, S7, S10–S17); and comparison of draw solutes (Table S1) (PDF)
