# Supplementary material for: Nanostructured Microsphere Production by Osmotic Extraction of Microfluidic Emulsion Templates
Source: Langmuir. 2025 Aug 6;41(32):21780–9. doi: 10.1021/acs.langmuir.5c02866 (PMC12368995; doi:10.1021/acs.langmuir.5c02866)
Supplement: Supplementary file 1 [file la5c02866_si_001.pdf]

Supporting Information for Nanostructured microsphere production by osmotic extraction of microfluidic emulsion templates

Kate A. Sanders, Michael F. L. De Volder**

kas89@cam.ac.uk; mfld2@cam.ac.uk

Department of Engineering, University of Cambridge, 17 Charles Babbage Road, Cambridge CB3 0FS

Contents:

Supplementary **Figures S1-S17**

Supplementary **Table S1**

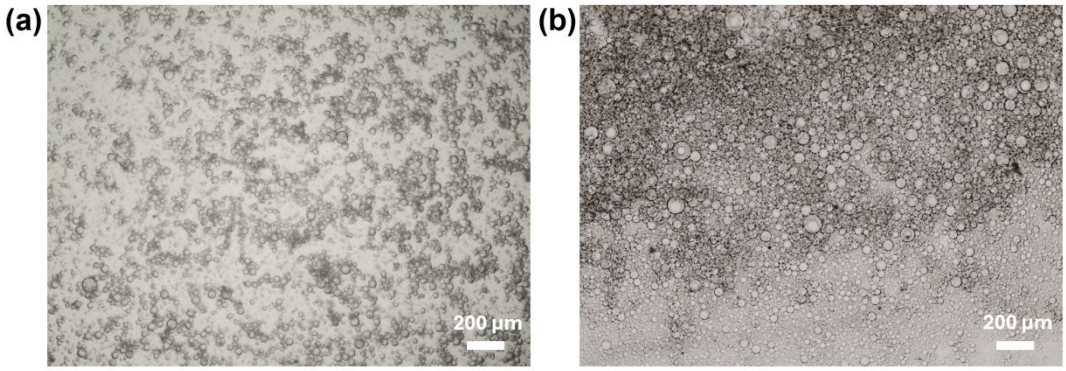

Figure S1: Transmission optical microscopy of coarse water-in-oil emulsion droplets emulsified by vortex mixing 1:10 (v:v) aqueous: oil phases at 2500 rpm for 30 seconds. (a) The aqueous phase was DI water (b) the aqueous phase was 3.2 M glucose. Samples were prepared for imaging by diluting in a thin layer of the oil phase.

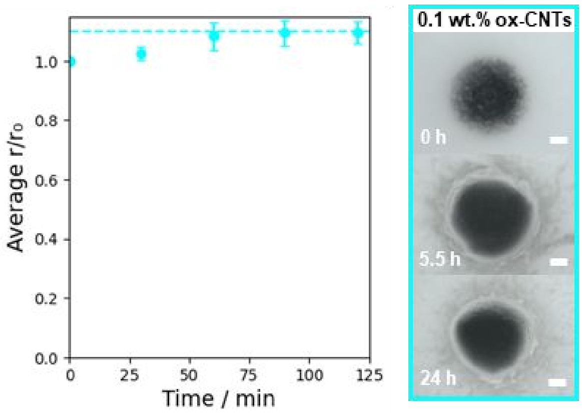

Figure S2: Template droplet behaviour in a 'draw' emulsion which contained no solute, only DI water. The change in average relative template droplet radius (r/r_0, n = 3), with time is shown on the left, and representative dark field optical micrographs are shown on the right. The initial template droplet radius, r_0, was around 500 µm; dashed lines indicate average r/r_0 after 24 h. Scale bars are 200 µm.

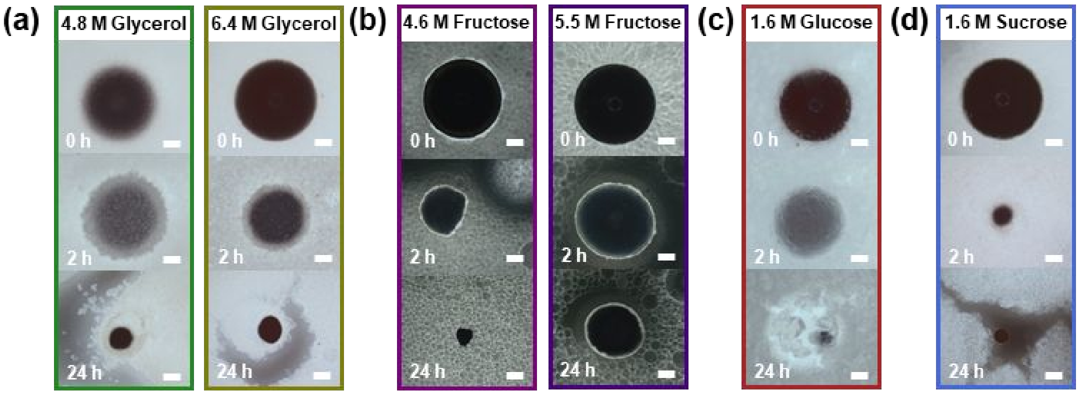

Figure S3: Representative dark field (DF) optical micrographs of template droplets containing 0.1 wt.% ox-CNT/H_2O in draw emulsions of varying composition. Draw solutes are: (a) 4.8 and 6.4 M glycerol (b) 4.6 and 5.5 M fructose (c) 1.6 M glucose; (d) 1.6 M sucrose. Scale bars are 200 μm.

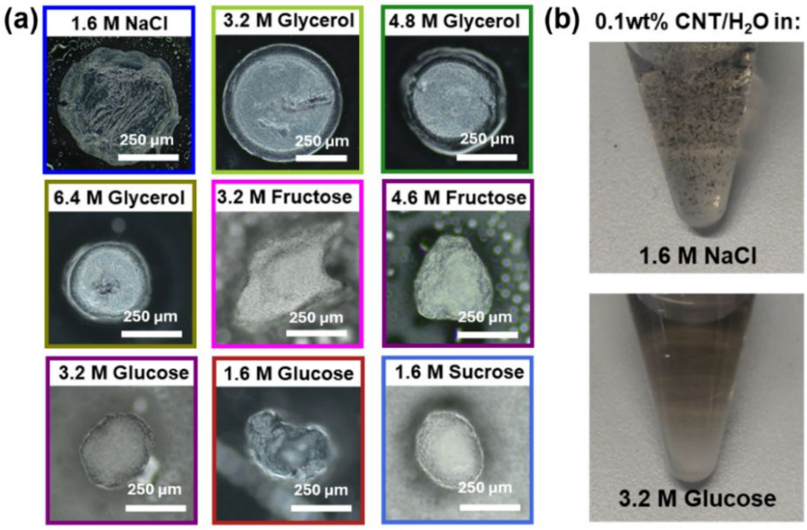

Figure S4: (a) DF optical micrographs of ox-CNT aggregates extracted from draw emulsions after 24 h and deposited on a glass slide to assess solidification. (b) aqueous draw solutions containing 1 drop of 0.1 wt.% ox-CNT/H_2O dispersion showing CNT aggregation upon contact with salt.

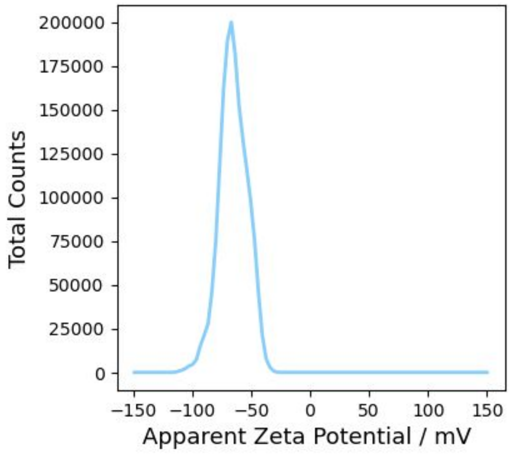

Figure S5: Zeta potential distribution of ox-CNTs dispersed in water (0.01 wt.% for characterisation). The apparent zeta potential was -65 ± 12 mV.

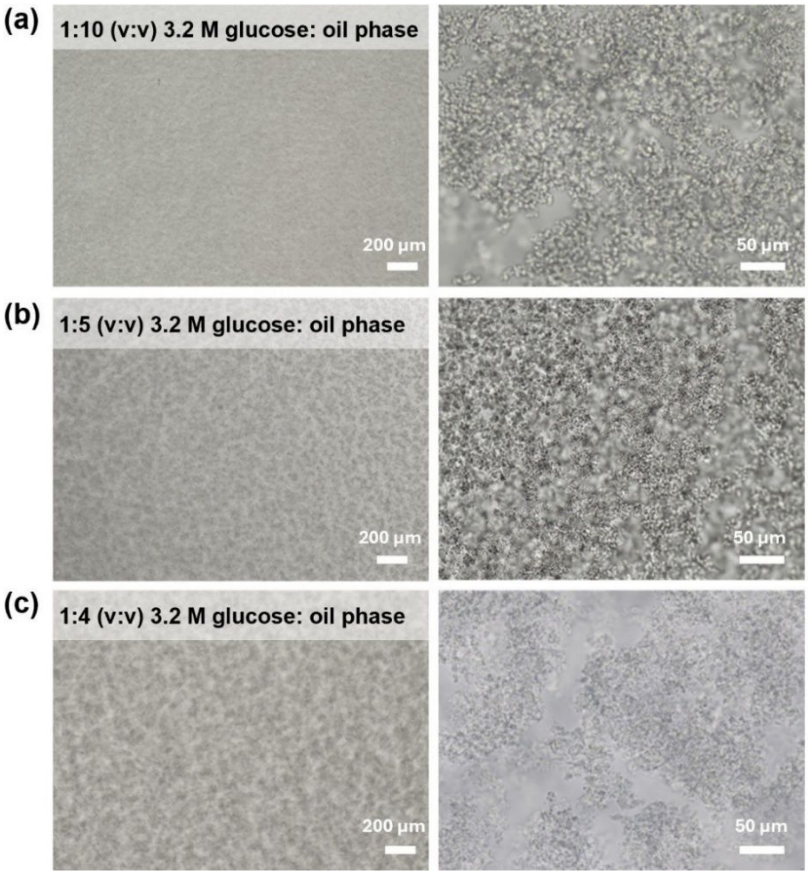

Figure S6: Transmission optical micrographs of fine draw emulsions generated by ultrasonication containing different glucose:oil ratios where: (a) 1:10; (b) 1:5; (c) 1:4.

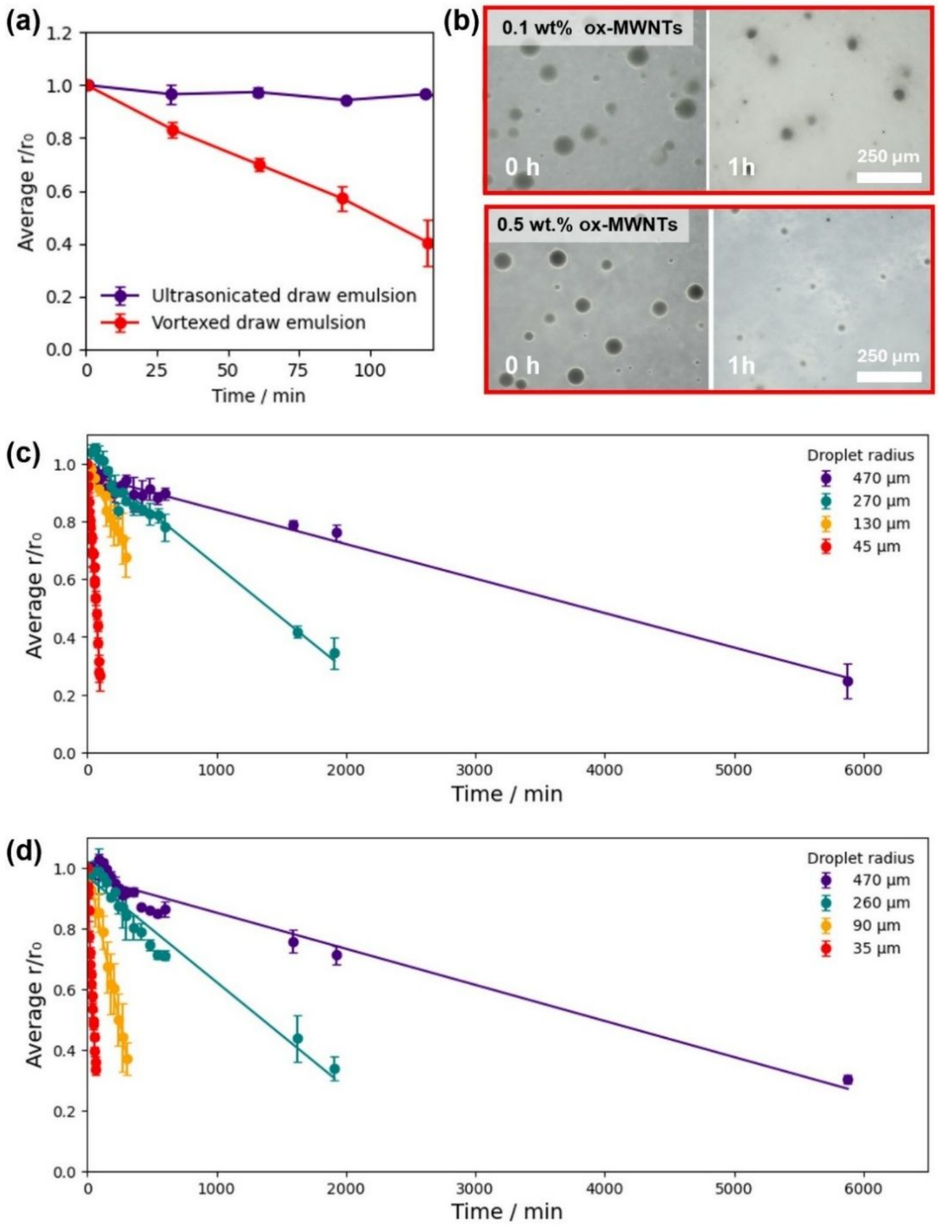

Figure S7: (a) Side by side comparison of ultrasonicated and vortex draw emulsion water extraction, showing the relative shrinkage rate of template droplets (n = 3) containing 0.1 wt.% ox-CNT/H_2O, with r_0 of around 500 µm, over 2 hours. (b) Representative DF optical microscopy of droplets with $r_0 < 200$ µm in diameter used for measurement of draw emulsion extraction rate. Only droplets which had no neighbours within a distance of 2x their initial radius were measured to limit interference. (c) The average relative shrinkage rate of different template droplet sizes containing 0.1 wt.% ox-CNTs in an ultrasonicated draw emulsion, as for Figure 4d but with a linear scale of time. (d) as for (c) but for 0.5 wt.% ox-CNT/H_2O template droplets, see Figure 4e in main text.

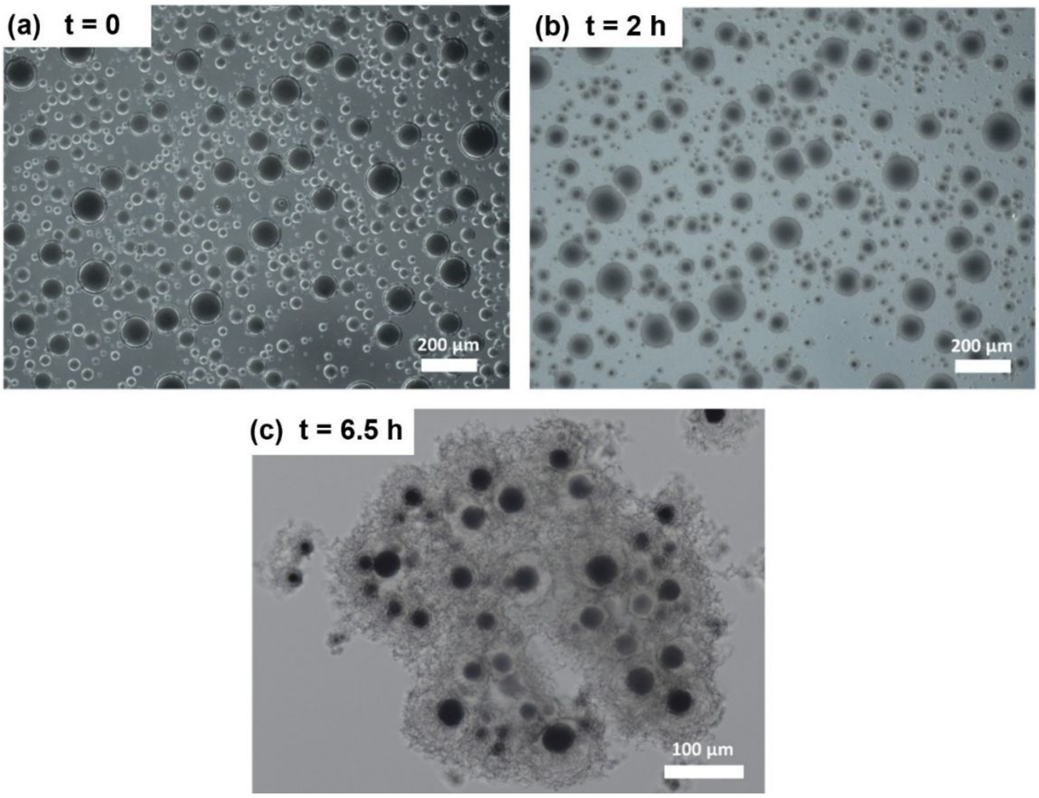

Figure S8: DF optical micrographs showing slow shrinkage of vortexed (bulk emulsified) W/O emulsion droplets containing 0.1 wt.% ox-MWNT/H_2O, without any draw emulsion present. (a) Droplets immediately after emulsification; (b) the same area after 2 h. The average r/r_0 of three selected droplets, where $r_0 = 52 \pm 2$ µm, was 0.71 after this time. (c) Transmission optical microscopy of flocculated regions and wet template droplets in the same emulsion after 6.5 h.

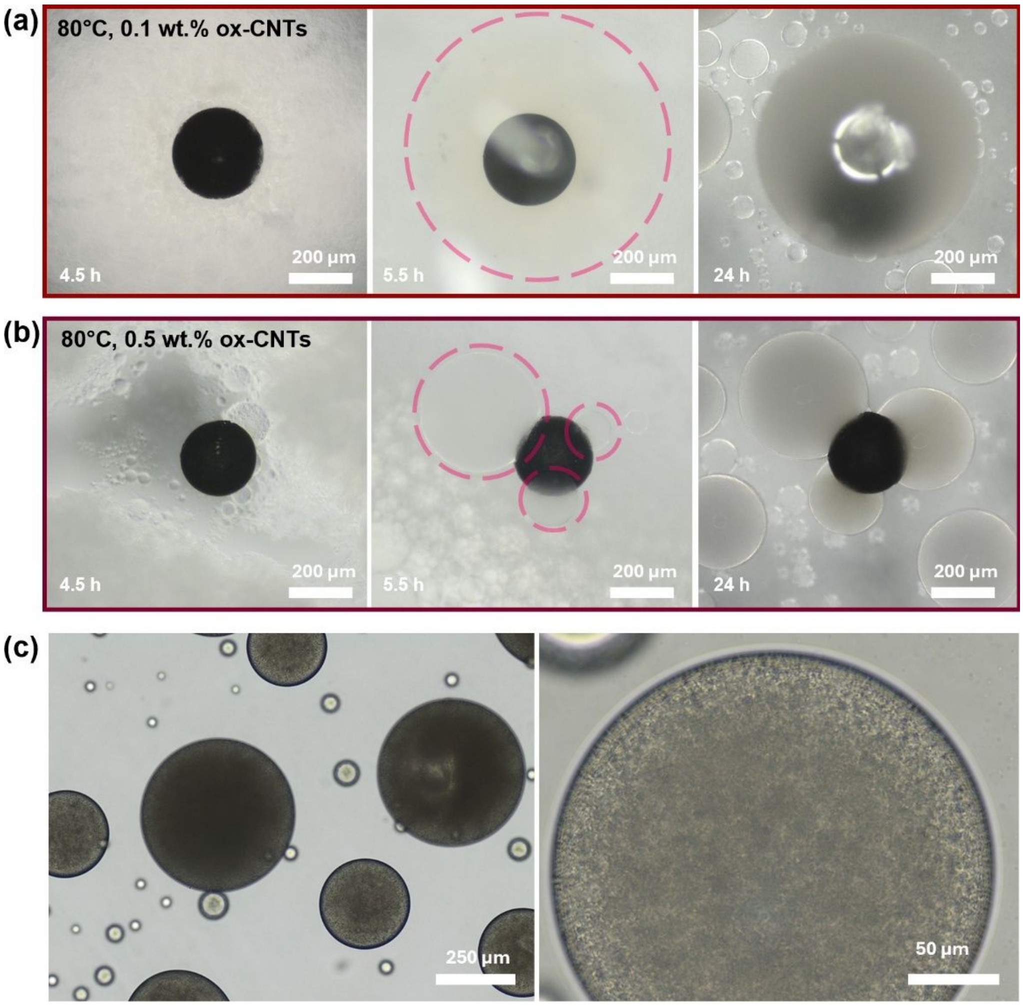

Figure S9: Optical micrographs showing 1:10 (v:v) 3.2 M glucose: oil draw emulsions heated to 80°C. (a, b) Magnified DF images of (a) 0.1 and (b) 0.5 wt.% ox-CNT containing template droplets in glucose-containing draw emulsions from 4.5 to 24 hours after emulsification. Dashed outlines show droplets which have coalesced with the developing CNT microparticle. (c) Transmission images of destabilised draw emulsion droplets, after 24 h. Large droplets with a complex internal structure are present, which may be due to the phase inversion behaviour of the ethoxylated surfactant Tween 85 at this temperature.

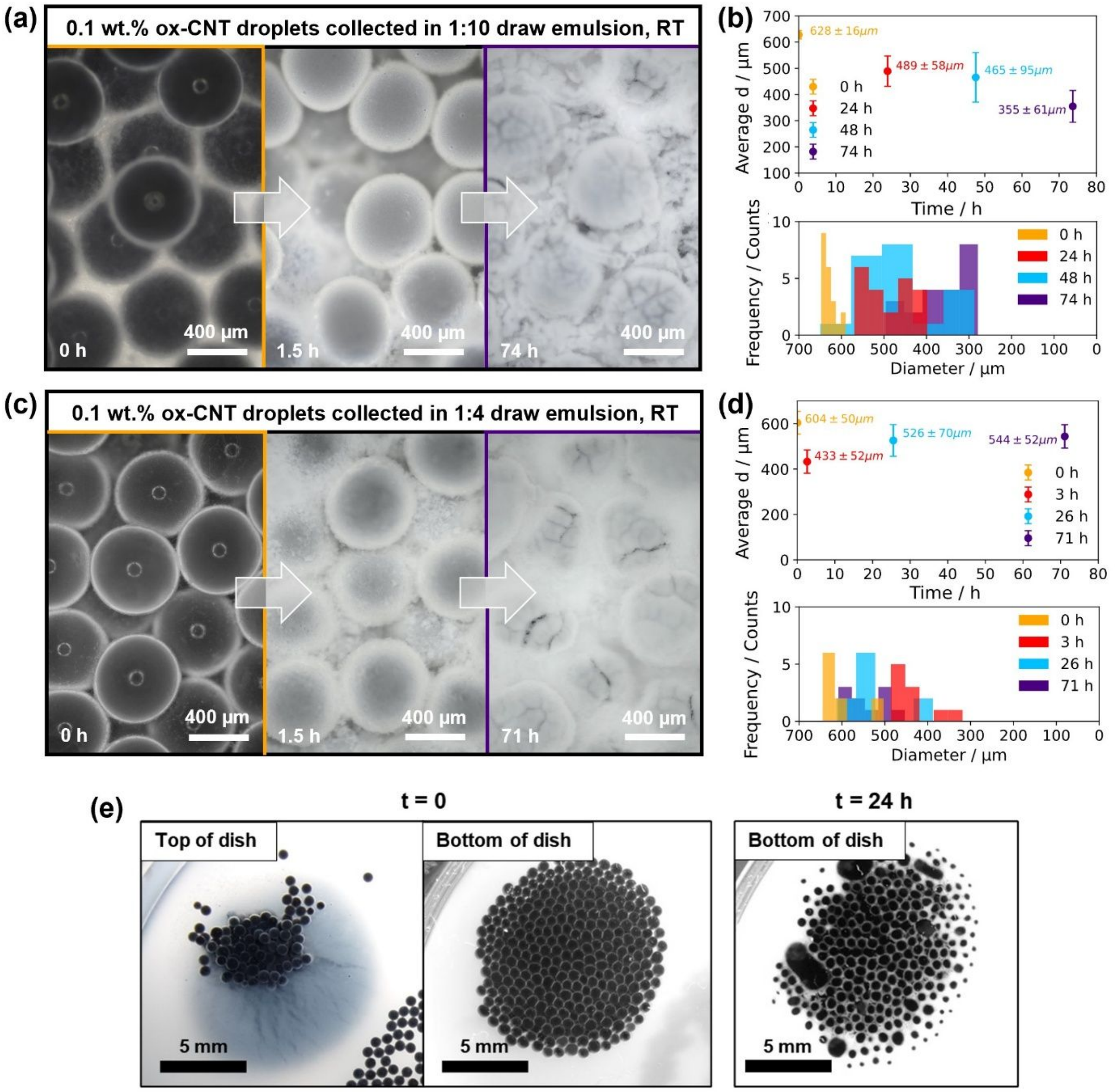

Figure S10: Microfluidic emulsified template droplets containing 0.1 wt.% ox-CNTs, emulsified in oil and collected in draw emulsions after exiting the device. (a) Dark field optical microscopy of droplets at room temperature after collection in a 1:10 glucose: oil phase draw emulsion. (b) measured average template droplet diameter (top, n = 20) and size distribution (bottom) of the emulsion in (a) over time. (c) As for (a) but using a 1:4 glucose: oil phase draw emulsion. (d) measured average template droplet diameter (top, n = 10 due to coalescence) and size distribution (bottom) of the emulsion in (c) over time. (e) photographs of poor mixing between draw and template droplet phases for the emulsion in (b), showing coalescence due to droplet compression.

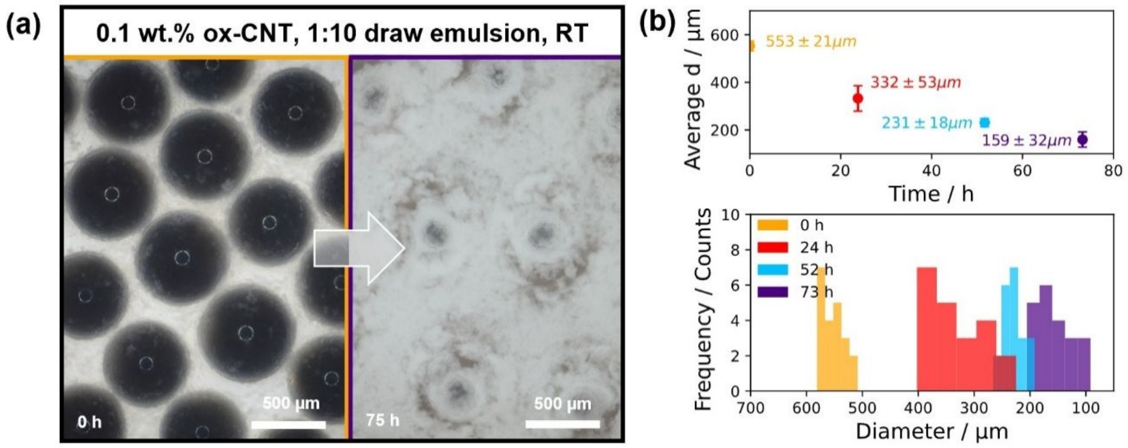

Figure S11: Osmotic pressure-driven solidification of microparticles at room temperature in 1:10 draw emulsions. (a) DF optical micrographs of microfluidic generated template droplets containing 0.1 wt.% ox-CNTs in water, undergoing solidification in a 3.2 M glucose draw emulsion with a 1:10 (v:v) glucose phase: oil phase composition. (b) measured average template droplet diameter (top, n = 20) and size distribution (bottom) of the emulsion in (a) over time.

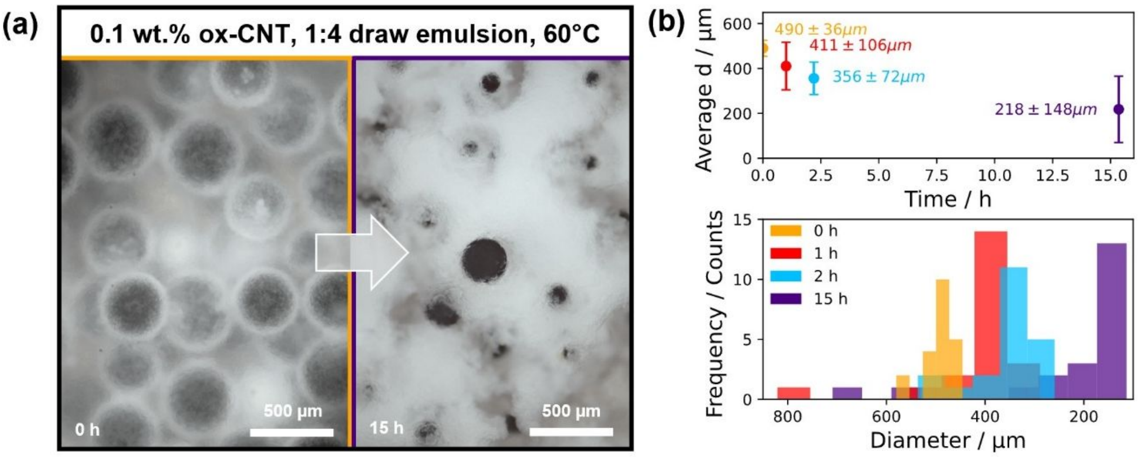

Figure S12: Destabilisation of batches of emulsions undergoing osmotic pressure-driven solidification at 60°C in a 1:4 v:v draw emulsion. (a) DF optical micrographs of microfluidic generated template droplets containing 0.1 wt.% ox-CNTs in water, undergoing solidification in a 3.2 M glucose draw emulsion with a 1:4 (v:v) glucose phase: oil phase composition. (b) measured average template droplet diameter (top, n = 20) and size distribution (bottom) of the emulsion in (a) over time, which broadens due to droplet coalescence.

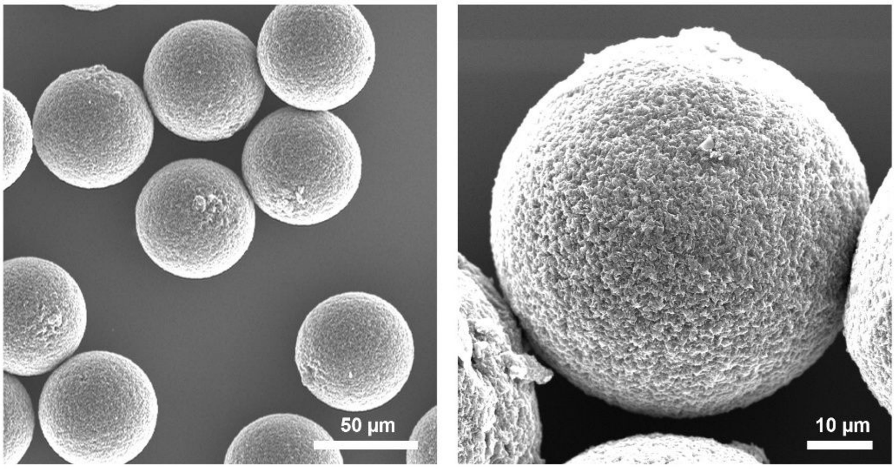

Figure S13: SEM images of CNT microparticles generated by microfluidic emulsification of 0.1 wt.% ox-CNT aqueous dispersions after solidification for 10 days at room temperature.

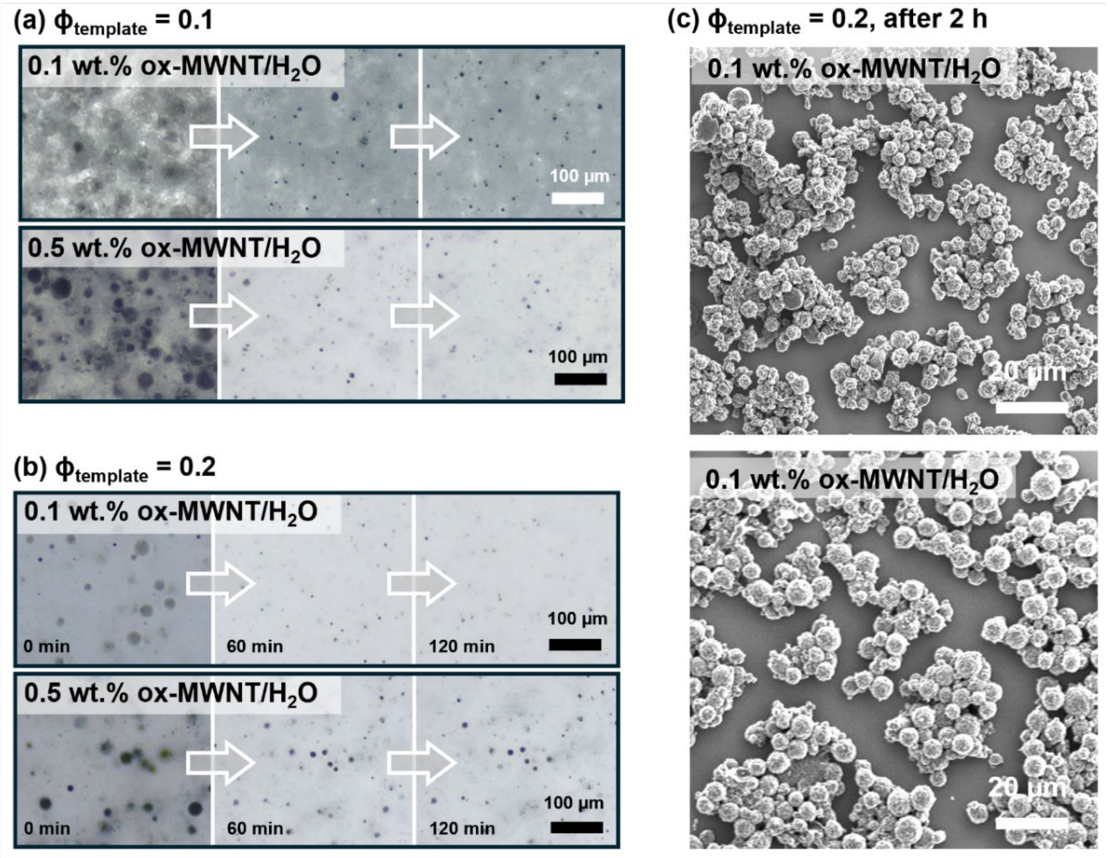

Figure S14: (a) DF optical micrographs showing the progressive shrinkage of aqueous polydisperse droplet templates containing ox-CNTs which were emulsified in oil, followed by mixing with draw emulsions to yield a 1:10 v:v glucose: oil phase total concentration, and template droplet volume fraction of $\phi = 0.1$. (b) as for (a) but where the template droplet volume fraction was 0.2. (c) SEM micrographs of CNT microparticles after 2 hours solidification in the draw emulsions in (b). The particles corresponding to those dried in (a) are shown in Figure 5 of the main text.

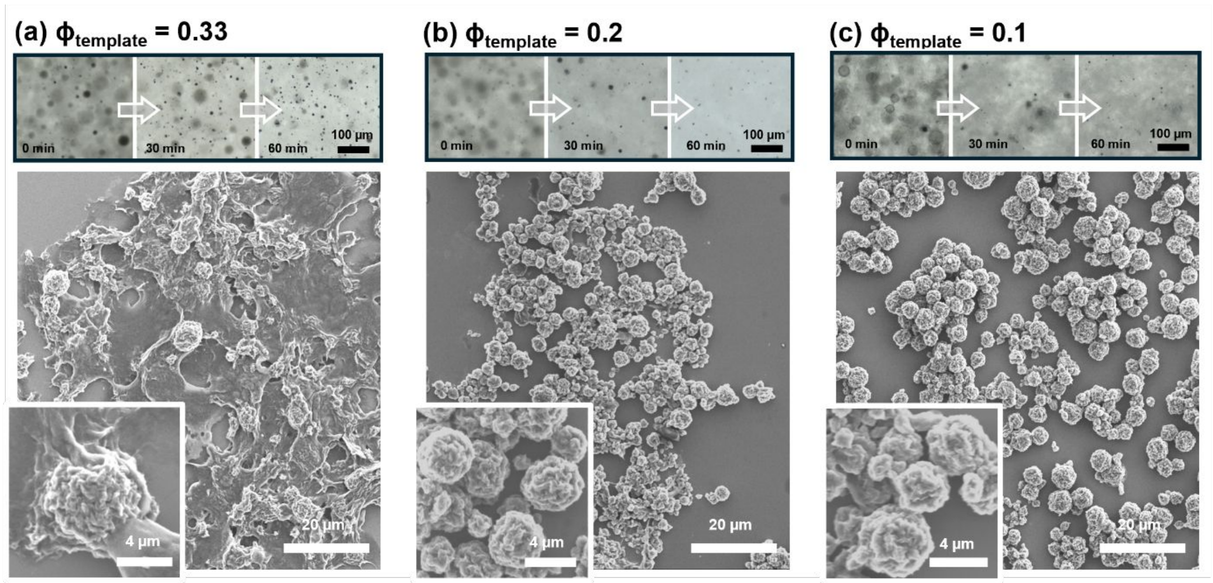

Figure S15: DF optical micrographs (top) and SEM images (bottom) showing the progressive solidification of CNT microparticles from 0.1 wt.% ox-MWNT/H2O dispersions after direct emulsification in 1:10 glucose: oil draw emulsions, with template droplet volume fractions of: (a) ϕ = 0.33; (b) 0.2; (c) 0.1. SEM images were taken after 1 h solidification time.

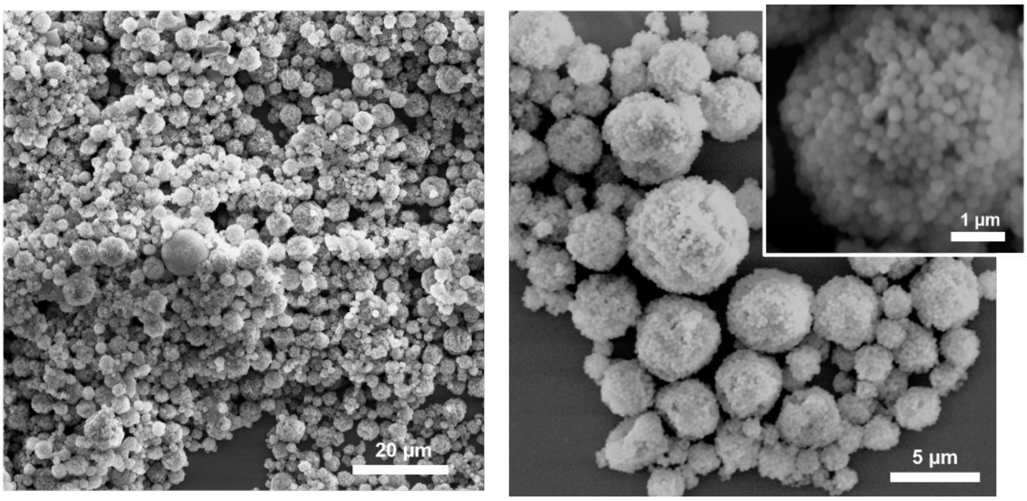

Figure S16: SEM images of microparticles assembled from silica nanoparticles through osmotic extraction processing of vortexed template droplets. The nanoparticles were synthesized by the Stober process, and had an average size of 285 ± 70 nm (DLS). An aqueous dispersion of 0.5 wt.% silica was emulsified at a template droplet volume fraction of 0.2. Droplet templates were mixed with a draw emulsion with 3.2 M glucose as the draw phase to yield a 1:10 glucose: oil phase ratio.

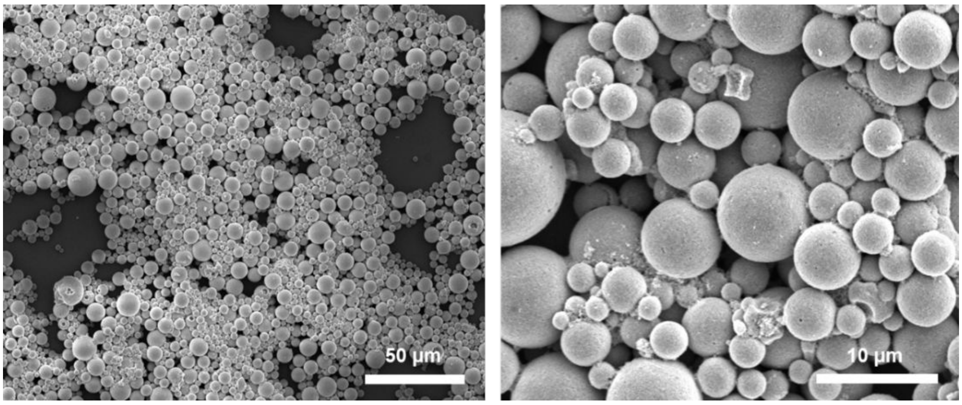

Figure S17: SEM images of microparticles assembled from lithium titanate nanoparticles through osmotic extraction processing of vortexed template droplets. Commercial spinel $Li_4Ti_5O_{12}$ nanoparticles with an average particle size of <200 nm were used as primary nanoparticles. An aqueous dispersion of 2 wt.% solid content was used, which was emulsified with a template droplet volume fraction of 0.2. Droplet templates were mixed with a draw emulsion with 3.2 M glucose as the draw phase to yield a 1:10 glucose: oil phase ratio.

Table S1: Summary of the aqueous draw solution properties and results of draw solute screening for the solidification of template droplets into ox-CNT microparticles by osmotic extraction.

Draw solute	Draw solution properties						Qualitative draw emulsion coalescence stability	$-d(r/r_0)/dt$ (linear fit for $r/r_0 > 0.3$ / s^{-1}	Average r/r_0 after 24 h	Solid particles after 24 h (washed) ?
	M / mol/L	m / mol/kg	ρ / kg/L [lit]	φ_m [lit]§	Osmolality/ osm/kg (calc.)	Osmotic pressure, Π / MPa (calc.)				
NaCl	1.6	1.7	1.06[1]	0.96[2]	3.3	8.6	OK	3.1×10^{-4}	0.85 ± 0.09	Flat, buckled
Urea	3.2	3.8	1.03[3]	0.90[2]	3.4	8.7	OK	N/A	0.98 ± 0.02	No, dissolved
Glycerol	3.2	4.1	1.07[1]	1.04[2]	4.3	11.3	Good	1.2×10^{-3}	0.38 ± 0.01	Flat, wet/sticky edges
	4.8	7.2	1.10[1]	1.06[2]	7.6	20.8	OK	1.1×10^{-3}	0.32 ± 0.02	Flat, wet/sticky edges
	6.4	11.6	1.14[1]	1.07[2]	12.4	35.1	OK	2.5×10^{-3}	0.31 ± 0.01	Flat, wet/sticky edges
Fructose*	3.2	5.0	1.21[1]	1.05[*4]	5.3*	15.7*	OK	1.4×10^{-3}	0.35 ± 0.02	Yes, spherical
	4.6	9.6	1.30[5]	N/A*	10.1*	32.5*	Poor	2.3×10^{-3}	0.22 ± 0.03	Flat, wet/sticky edges
	5.5	14.9	1.35 (est.)[5]	N/A*	15.6*	52.3*	Very poor	1.3×10^{-3}	0.54 ± 0.16	No
Glucose	3.2	5.0	1.21[1]	1.13[6]	5.7	16.9	Good	4.8×10^{-3}	0.22 ± 0.01	Yes, spherical
	1.6	2.0	1.10[1]	1.03[6]	2.1	5.6	Good	0.16×10^{-3}	0.34 ± 0.15	Yes, spherical
Sucrose	2.4	5.0	1.30[1]	1.45[2]	7.3	23.4	Very poor	18×10^{-3} (30 min)	0.32 ± 0.07	No, dissolved
	1.6	2.5	1.20[1]	1.23[2]	3.0	9.2	OK	5.5×10^{-3}	0.21 ± 0.03	Yes, spherical

§ Literature values of φ_m are reported at 25°C with the exception of fructose.

* There is very limited literature data available on the osmotic coefficient of concentrated aqueous fructose solutions. The molal osmotic coefficient for a 5.4 mol/kg solution at 308.15 K has been experimentally determined as $\varphi_m = 1.05$. This value of φ_m was used to provide an approximate estimate of the osmolarity and osmotic pressures of the three concentrated fructose draw solutions tested in this work.

References:

(1) Haynes, W. M., Lide, D. R., Bruno, T. J. *CRC Handbook of Chemistry and Physics*, 97th ed.; Taylor & Francis Group, 2017.

(2) George Scatchard, B.; Hamer, W. J.; Wood Sinclair, S. E.; Sinclair, D. A.; Robinson, R. A. Isotonic Solutions. I. The Chemical Potential of Water in Aqueous Solutions of Sodium Chloride, Potassium Chloride, Sulfuric Acid, Sucrose, Urea and Glycerol at 25°. *J Am Chem Soc* **1938**, *60* (12), 3061–3070.

(3) Halonen, S.; Kangas, T.; Haataja, M.; Lassi, U. Urea-Water-Solution Properties: Density, Viscosity, and Surface Tension in an Under-Saturated Solution. *Emission Control Science and Technology* **2017**, *3* (2), 161–170. https://doi.org/10.1007/s40825-016-0051-1.

(4) Ebrahimi, N.; Sadeghi, R. Osmotic Properties of Carbohydrate Aqueous Solutions. *Fluid Phase Equilib* **2016**, *417*, 171–180. https://doi.org/10.1016/j.fluid.2016.02.030.

(5) *D-Fructose density concentration table*. Mettler Toledo. https://www.mt.com/my/en/home/supportive_content/concentration-tables-ana/D_Fructose_de_e.html (accessed 2021-03-20).

(6) Bonner, O. D.; Breazeale, W. H. Osmotic and Activity Coefficients of Some Nonelectrolytes. *J Chem Eng Data* **1965**, *10* (4), 325–327.
